# Current Best Evidence for 5 Promising Medications Used for Scar Minimization Therapy

**DOI:** 10.1177/22925503251322528

**Published:** 2025-03-13

**Authors:** Nickesh Dua, Emily Burke, Jack Rasmussen

**Affiliations:** 1 Dalhousie Medical School, Dalhousie University, Halifax, Nova Scotia, Canada; 2 Division of Plastic Surgery, Department of Surgery, Halifax, Nova Scotia, Canada; 3 Department of Critical Care Medicine, Dalhousie University, Halifax, Nova Scotia, Canada

**Keywords:** scar, fibrosis, antifibrotic, cutaneous scarring, Mots-clés, Cicatrice, fibrose, antifibrotique, cicatrisation cutanée

## Abstract

**Introduction:** Wound healing by fibrosis allows for closure of a wound, but leaves behind a permanent scar with physical and psychological effects. The primary aim of this narrative review was to summarize the current status of the evidence supporting the use of oral or topical medications to minimize scarring in humans. **Methods:** With the help of a health sciences librarian, PubMed, Embase, and Scopus were searched up to March 31, 2023, to investigate potential medications to ameliorate scarring. Based on this search, the medications pirfenidone, losartan, trichostatin A, enalapril, and atorvastatin were identified as 5 therapies with the most research to support their use. Studies discussing noncutaneous scarring (myocardial, intraabdominal, etc) or in animal models were excluded. **Results:** There is a paucity of quality literature describing the use of oral or topical medications to minimize fibrosis and produce more favorable scarring. Six studies describing the medications listed above all demonstrated an improvement in scarring parameters, most commonly based on the Vancouver Scar Scale. **Conclusions:** Though preliminary, emerging evidence suggests that therapies already exist with the potential to improve cutaneous scarring. Some of these medications are already ubiquitous, affordable and have a known safety profile. Excitingly, these treatments are either oral or topical, meaning that they are more accessible for patients than some current modalities for scar treatment, including steroid injections or laser therapy. Further larger-scale trials are needed before these treatments can be recommended as a routine part of scar management.

## Résumé

**Introduction :** La fibrose permet à une plaie de se fermer et de guérir, mais laisse derrière elle une cicatrice permanente aux conséquences physiques et psychologiques. Le but principal de cette revue narrative était de résumer l’état actuel des données étayant l’utilisation des médicaments oraux ou topiques visant à minimiser les cicatrices chez les humains. **Méthodes :** Des recherches ont été menées dans les bases de données PubMed, Embase et Scopus avec l’aide d’un bibliothécaire en sciences de la santé, jusqu’à la date du 31 mars 2023 pour étudier les médicaments susceptibles d’améliorer la cicatrisation. La pirfénidone, le losartan, la trichostatine A, l’énalapril et l’atorvastatine ont été identifiés dans cette recherche comme étant les traitements ayant fait l’objet du plus grand nombre d’études étayant leur utilisation. Les études traitant de cicatrisation non cutanée (myocardique, intra-abdominale, etc.) ou sur des modèles animaux ont été exclues. **Résultats :** Il y a très peu d’articles de qualité décrivant l’utilisation de médicaments topiques ou administrés par voie orale dans le but de réduire la fibrose et d’aboutir à une cicatrisation plus favorable. Six études décrivant les médicaments décrits ci-dessus ont toutes montré une amélioration des paramètres de cicatrisation reposant le plus souvent sur l’échelle de cicatrisation de Vancouver. **Conclusion :** Bien que préliminaires, les données probantes émergentes suggèrent que des traitements susceptibles d’améliorer la cicatrisation cutanée existent déjà. Certains de ces médicaments sont déjà largement disponibles, peu dispendieux et ont un profil de sécurité connu. Il est aussi très intéressant de noter que ces médicaments s’utilisent par voie orale ou topique et qu’ils sont plus accessibles aux patients que certaines modalités actuelles du traitement des cicatrices impliquant des injections de corticostéroïdes ou une thérapie laser. D’autres essais à plus grande échelle sont nécessaires avant de pouvoir recommander ces traitements dans le cadre de la gestion usuelle des cicatrices.

## Introduction

The process of fibrosis, while important for wound closure, restoration of function, and provision of a matrix for remodeling, is not perfect. It yields a visible reminder of the events associated with the initial wound which may lead to psychological effects and psychosocial barriers^1^ for the patient. Additionally, the process fails to restore sebaceous glands and hair follicles, which may lead to significant functional impairments, and a reduction in the tensile strength to an overall maximum of 80% of that of normal skin.^2^ Thus, from several standpoints, reducing the resultant scar could have profound benefits for the individual.

Over-the-counter treatments aimed at reducing the appearance of scars are widely available, but their use and efficacy are not backed by high-quality research. Furthermore, their benefit is limited, as their mode of action is typically mechanical in nature and largely affects the scar after it has already formed, rather than altering the internal physiologic process of fibrosis in scar formation and remodeling. A more targeted approach with pharmacological interventions that encourage healing with less of the deleterious effects of fibrosis and more closely mimics healthy skin is desirable.

Inhibition of scarring pathways first requires background knowledge of the process itself. Once dermal tissue is damaged, the coagulation cascade is initiated which results in the formation of a fibrin clot. Inflammation ensues to recruit specialized cells such as macrophages to the site of injury to remove cellular debris, prevent infection, and remove fibrin.^3^ The subsequent proliferative phase is the focus of this investigation, whereby fibroblasts migrate to the site to lay down connective tissues, including fibrillary collagen types I, III, and V, basement collagens (type IV), and noncollagenous matrix molecules, such as fibronectin, fibrillin, elastin, and proteoglycans.^4^ Deposition of collagens (primarily collagen III) and proteoglycans leads to a densely packed but comparatively weaker tissue, to later be remodeled and replaced with collagen I.^5^ The purpose of scarring is to replace the tissue defect and restore tissue cohesion. During remodeling, the fibroblasts either retreat or undergo apoptosis.^5^ Concurrent with this remodeling, the microvasculature which has developed in the scar also matures, and the initial red/pink discoloration slowly fades into a paler color, corresponding with the level of vascularization.^6^ Overall, the remodeling phase may last up to one year, after which point the mature scar will largely remain unchanged.^3^ Theoretically, during the proliferative and remodeling phases a scar may be amenable to interventions to allow it heal more like normal skin and less like scar tissue.

In order to ascertain the current evidence of antifibrotic medication on scar outcomes and identify potential next steps, a review of the existing literature was performed. Our aim is to summarize the current understanding of topical and oral medications that can ameliorate the process of fibrosis, stimulate further research, and eventually provide clinicians with an approach to treating or decreasing patient scar formation, thus improving cosmesis and reducing negative outcomes associated with significant scarring.

## Methods

### Background Search

A preliminary search for medications with antifibrotic properties was conducted by searching PubMed, Embase, and Scopus. Based on the evidence in the literature, the medications pirfenidone, losartan, trichostatin A, enalapril, and atorvastatin were identified as having the most evidence to support their utility and were selected for a proper literature search. A search of commonly used synonyms for scar, scarring, and the antifibrotic process was conducted and yielded the terms for our search strategy: scar, scars, scarring, scarification, cicatrix, cicatrization, fibrosis, fibrose, fibrosing. Additionally, in the event a different spelling was utilized for a drug, such as “losartan” or “losartane” or “losartan s”; “atorvastatin” or “atorvastatine” or “atorvastatine s”; it was included in the search strategy as well to ensure all the available articles were captured. A professional medical sciences librarian assisted with our literature search and to select appropriate terms for our search strategy.

### Search Strategies

The database search involved utilizing PubMed, Embase, and Scopus with the appropriate search terms and syntax for the specific databases. The exact search terms are found in Appendix 1.

### Literature Search

The search terms were applied to the databases and executed/updated to March 31, 2023. The results from each database were extracted, and imported into Covidence for deduplication and streamlining of the search. The total number of papers from the search yielded 946 studies. There were 284 duplicates removed giving a final total of 662 studies. Inclusion criteria were applied to the literature, stating that the paper must involve the medications listed, involve cutaneous scarring, and include an objective measurement for comparison; animal studies were included to provide mechanistic background information and proof of concept. Exclusion criteria were any papers discussing nonepidermal scarring (such as intra-abdominal, myocardial, and intraspinal). The initial screen yielded 55 studies which were subsequently examined further, with stricter exclusion criteria such as abstract only, presentation only, case reports, or commentary pieces; this yielded a final total of 39 studies that were included, with both human and animal models involved. Ultimately, only 6 human studies met the full inclusion and exclusion criteria and were utilized for comparison of medication efficacy. The remaining articles involving animal studies were utilized for background information and/or mechanistic insight. Appendix 2 summarizes the PRISMA results from Covidence.

## Results

### Pirfenidone

Pirfenidone is an antifibrotic agent initially used for the treatment of pulmonary fibrosis. It works by decreasing fibroblast activity, inhibiting collagen fibril deposition, and lowering TGF-β levels.^7^ Armendariz-Borunda et al conducted a controlled clinical trial on pediatric burn patients aged 3 to 16 years old with hypertrophic scars using an 8% pirfenidone topical gel 3 times a day for 6 months and pressure therapy on 33 patients versus 30 patients with similar scars treated with pressure therapy alone.^8^ They found that 9 of 33 patients in the experimental group (27%) had a decrease in their Vancouver Scar Scale (VSS) scores by more than 55%, 22 patients (67%) had a 30% to 45% decrease, whereas 2 patients (6%) had a 30% decrease or less. The control group was treated with pressure therapy only and showed a slight improvement of 16% in VSS score on average.^8^

### Topical Angiotensin II Receptor Blocker: Losartan

Angiotensin II (ATII) is a pro-inflammatory molecule in a healing wound; Angiotensin receptor blockers (ARBs) seem to reduce scarring by decreasing VEGF levels, decreasing collagen production, and inhibiting mast cell release.^9^ Hedayatyanfard et al conducted a single-blind study with a prepared 5% by weight losartan ointment and compared it with placebo ointment.^9^ Participants used the ointment for 3 months on scars less than 6 months in age, and after removing drop-outs from their study, they had 20 participants in the treatment group compared with 10 participants in the placebo group. Their scars were assessed with the VSS, as well as self-reported pain and itching measures. The group found a reduction from 8 to 5.5 points on the VSS, compared to 0.5/negligible change within margin of error with placebo in keloid scars, and found a 3-point reduction in hypertrophic scars on the VSS compared to no change in the placebo group.^9^

Khodaei et al conducted a double-blind controlled trial on 24 female patients with surgical wounds for cosmetic mammoplasty or abdominoplasty, using a 5% losartan ointment on one portion of the scar compared to placebo on another portion.^10^ The participants’ scars were assessed with the VSS. The team found that the treated scars had statistically reduced mean VSS scores: 7.1 ± 2.06 (3 months) and 5.21 ± 1.71 (6 months) compared to placebo 9.77 ± 1.55 (3 months) and 8.31 (6 months); there was a difference of 2.67 at 3 months and 3.1 at 6 months, respectively. They also noted that the treated sides also had reduced total VSS, and reduced VSS subsets including height, pliability, and vascularity.^10^

### Topical Angiotensin-Converting Enzyme Inhibitors: Enalapril

Angiotensin-converting enzyme (ACE) converts angiotensin I to its active form, ATII. As described above, ATII seems to increase fibroblast proliferation and collagen production. By inhibiting ACE activity, angiotensin-converting enzyme inhibitor (ACEI) has been shown to decrease both cutaneous and organ scarring after injury.^11^ In a double-blind controlled trial, Mohammadi et al compared using a 1% enalapril ointment with placebo on 30 patients with second or third-degree burns over a 6-month period.^11^ They assessed scars by measuring size and thickness monthly, and ascertained patients’ self-reported itching scores on a scale between 0 (no itching) and 4 (worst itching possible). The team found that the Enalapril-treated group had smaller scars at 2.02 ± 0.55 compared with controls, which had 2.30 ± 0.64. Unfortunately, units of measurement were not reported. It was also noted that itching was lower in the treated group with a mean self-reported score of 1.73 ± 0.69 compared with the controls’ average score of 2.43 ± 0.67.^11^

### Oral ACEIs or ARB

Hu et al conducted a questionnaire-based observational study on 347 patients with postop thyroidectomy scars.^12^ The group asked patients taking an oral ACE inhibitor or ARB to select the medication they were on from a comprehensive list. All scars in the study were photographed and then evaluated using the Scar Cosmesis and Rating scale to maintain the evaluators blind to the subjects. They found that scar width of both patients taking an ACEI (1.60 ± 0.30 mm [95% CI: 1.46-1.73]) or ARB (1.57 ± 0.45 mm [95% CI: 1.38-1.76]) were narrower than the antihypertensive control (patients on a non-ACEI/ARB antihypertensive medication) group (2.09 ± 0.79 mm [95% CI: 1.82-2.37]) and the blank control group (2.00 ± 0.93 mm [95% CI: 1.75-2.26]), respectively.^12^

### Statins: Atorvastatin

Statin medications seem to inhibit excessive scar formation via a variety of mechanisms, including decreasing receptors for ATII to bind to, as well as reducing oxidative stress and inflammation.^13^ Chello et al conducted a retrospective study of 930 cardiac surgery patients and calculated atorvastatin equivalent dose of the statin patients were utilizing.^14^ The participants’ scars were evaluated by a dermatologist using the VSS height variable only, to diagnose a hypertrophic scar. They found a lower incidence of hypertrophic scars in statin users, and a proportional decrease in their incidence was observed with increasing statin dose; 79.8% of the patients in this study who took the various dosages of atorvastatin had a normal wound healing process. Logistic regression analysis confirmed the protective role of statins after adjustment for age. The dose-dependent effect was confirmed in regression analysis, showing a more intensive protective effect for higher doses of statins at 40 or 80 mg.^14^ The scars themselves were not analyzed with respect to VSS parameters. Chello et al indicated that the use of statins prevented the development of hypertrophic scars.

## Discussion

Based on our review of the literature, it appears in limited trials that pirfenidone, ACE inhibitors, ARBs, and statins have therapeutic benefits in reducing the size and visibility of scars, or in the case of statins at least reduce the likelihood of hypertrophic scarring. The benefit of ACE inhibitors, ARBs, and statins is that they are already used routinely for the treatment of hypertension and hypercholesterolemia, and as such are easily accessible, and have a well-recognized safety profile. They are also reasonably affordable, another key factor in making sure these are a practical option to help patients minimize excessive scarring. As such, these medications have the potential to significantly reduce the physical and psychological burden experienced by patients postinjury and postoperatively, especially when a patient's primary goal is scar minimization. By reducing the pro-fibrotic process, these medications allow healing to occur with less scar tissue formation, and thus also reduced scar tissue remodeling, yielding significantly better cosmesis. Improved cosmesis and tissue healing arguably should be included in postsurgical goals of care, and perhaps one-day utilizing antifibrotic therapies could become the standard of care alongside managing pain and infection.

A promising future direction includes the drug verteporfin which shows promising results in pigs and mice in full restoration of skin, including sebaceous gland and hair follicle, full tensile strength restoration, and no scarring^15^; clinical trials in humans are awaiting FDA approval in the United States. Although verteporfin is currently a medication delivered intravenously or injectable into the wound, next steps if successful could lead to an oral or topical formulation, which would make its application more widely accessible.

It is important to note that there remains a significant dearth of high-quality clinical research on available and easy-to-administer pharmacological interventions to impact fibrosis. While a tremendous amount of research has been conducted on the physiology of fibrosis and agents to impact it, the vast majority are in-vitro or animal-based. Trials investigating antifibrotic therapies in humans are sparse, and the trials listed above are single center, have relatively low treatment group numbers, or are very narrow in their scope. As such, there is not enough evidence to date to make any sort of clinical recommendations at this time with confidence. With that said, the promising results of the studies described suggest that larger, multicenter randomized control trials are warranted. Over the course of a lifetime, essentially every one of us will sustain a scar, either after injury or surgery, so the potential benefit of a safe, affordable, easy-to-use medication that has demonstrable benefits in scar formation would be significant (Table 1).

**Table 1.** Study Findings Summary.

Authors	Intervention	Wound type	Subject (n)	Findings summary
Armendariz-Borunda et al^8^	8% pirfenidone ointment & pressure therapy versus pressure therapy alone	Hypertrophic burn scars	33 (pirfenidone) versus 30 (control)	Significant improvement in VSS after 6 months of use (*P* ≤ .01) and significant improvement compared to control group (*P* ≤ .01)
Hedayatyanfard et al^9^	5% losartan ointment versus placebo ointment	Hypertrophic and keloid scars	20 (losartan) versus 10 (placebo)	Significant decrease in VSS scores in losartan group (*P* ≤ .01)
Khodaei et al^10^	5% losartan ointment versus placebo ointment	Cosmetic mammoplasty and abdominoplasty scars	24 subjects (self-controlled design)	Significant reduction in VSS scores at 3 and 6 months in losartan treated scars versus placebo
Mohammadi et al^11^	1% enalapril ointment versus placebo ointment	Hypertrophic scars after partial and full-thickness burns	30 patients (self-controlled design)	Significant reduction in size and thickness of scars in enalapril group after 6 months of treatment
Hu et al^12^	Patients taking oral ACEI or ARB versus those not taking ACEI or ARB	Thyroidectomy scars	347 patients (questionnaire-based study)	Significant reduction in scar width in patients taking either ACEIs or ARBs compared to control
Chello et al^14^	Patients taking oral statin versus patients not on statins	Sternotomy scars after cardiac surgery	930 patients (retrospective	Scar height component of VSS was significantly less in treatment group

Abbreviations: ACEI, angiotensin-converting enzyme inhibitor; ARB, angiotensin receptor blocker; VSS, Vancouver Scar Scale.

Our hope is that eventually there will be sufficient evidence to help clinicians and patients choose antifibrotic medications early in the healing process. Scar reduction therapies have a wide range of benefits including cosmetic appeal,^1^ restoration of function and strength,^13^ and also psychological well-being since many individuals have a traumatic event tied to their scar.^1^ We hope that future developments in medicine and surgery will make it possible to completely eliminate any evidence of scarring, which would be a dramatic advancement in the art and science of medicine.

## Supplemental Material

sj-docx-1-psg-10.1177_22925503251322528 - Supplemental material for Current Best Evidence for 5 Promising Medications Used for Scar Minimization Therapy Supplemental material, sj-docx-1-psg-10.1177_22925503251322528 for Current Best Evidence for 5 Promising Medications Used for Scar Minimization Therapy by Nickesh Dua, Emily Burke and Jack Rasmussen in Plastic Surgery
