# Supplementary material for: Current Best Evidence for 5 Promising Medications Used for Scar Minimization Therapy
Source: Plast Surg (Oakv). 2025 Mar 13;34(2):301–5. doi: 10.1177/22925503251322528 (PMC11907609; doi:10.1177/22925503251322528)
Supplement: sj-docx-1-psg-10.1177_22925503251322528 - Supplemental material for Current Best Evidence for 5 Promising Medications Used for Scar Minimization Therapy [file sj-docx-1-psg-10.1177_22925503251322528.docx]

| Database | Search Strategy |
| --- | --- |
| **PubMed** | ("pirfenidone"[Supplementary Concept] OR "pirfenidone"[tiab] OR "losartan"[MeSH Terms] OR "losartan"[tiab] OR "losartan s"[tiab] OR losartane[tiab] OR "atorvastatin"[MeSH Terms] OR "atorvastatin"[tiab] OR "atorvastatine"[tiab] OR "atorvastatin s"[tiab] OR "enalapril"[MeSH Terms] OR "enalapril"[tiab] OR "Trichostatin a"[tiab]) AND ("cicatrix"[MeSH Terms] OR cicatrix[tiab] OR Cicatrization[tiab] OR scars[tiab] OR scar[tiab] OR scarring[tiab] OR scarification*[tiab]) |
| **Scopus** | TITLE-ABS-KEY ( pirfenidone OR losartan OR "losartan s" OR "atorvastatin" OR "atorvastatine" OR "atorvastatin s" OR "enalapril" OR "Trichostatin a" ) AND TITLE-ABS-KEY ( cicatri* OR scars OR scar OR scarring OR scarification* ) |
| **Embase** | ('pirfenidone'/exp OR 'pirfenidone' OR 'atorvastatin'/exp OR 'atorvastatin' OR 'losartan'/exp OR 'losartan' OR 'enalapril'/exp OR 'enalapril' OR 'trichostatin a'/exp OR 'trichostatin a') AND ('scar'/exp OR scar OR scars OR cicatri* OR scarification OR scarring) AND [embase]/lim NOT ([embase]/lim AND [medline]/lim) |

**Appendix 1**

**Appendix 2**

| 946 references imported for screening as 946 studies  284 duplicates removed  662 studies screened against title and abstract  607 studies excluded  55 studies assessed for full-text eligibility  16 studies excluded  10 Abstract only  2 Wrong comparator  2 Wrong study design  1 Case report  1 Response to article with same title  0 studies ongoing  0 studies awaiting classification  39 studies included |
| --- |
